# Relation between Age-Related Macular Degeneration and Cardiovascular Events and Mortality: A Systematic Review and Meta-Analysis

**DOI:** 10.1155/2016/8212063

**Published:** 2016-12-14

**Authors:** Jie Wang, Yangjing Xue, Saroj Thapa, Luping Wang, Jifei Tang, Kangting Ji

**Affiliations:** Department of Cardiology, The Second Affiliated Hospital and Yuying Children's Hospital, Wenzhou Medical University, Xueyuanxi Road, No. 109, Wenzhou, Zhejiang 325000, China

## Abstract

Data on the association between age-related macular degeneration (AMD) and cardiovascular disease and mortality are conflicting. The purpose of this report is to conduct a systematic review to better understand the role of AMD as a risk factor for CVD events and mortality. We searched Medline (Ovid) and Embase (Ovid) for trials published from 1980 to 2015. We included 20 cohort studies that reported relative risks with 95% confidence intervals for the association of AMD and cardiovascular events and mortality, involving 29,964,334 participants. In a random-effects model, the adjusted RR (95% confidence interval [CI]) associated with AMD was 1.08 (1.00–1.117) for all-cause mortality (8 studies) and 1.18 (0.98–1.43) for cardiovascular disease mortality (5 studies). The pooled RR (95% CI) was 1.17 (0.94–1.45) for coronary heart disease (CHD; 3 studies) and 1.13 (0.93–1.36) for stroke (8 studies). Findings from this systematic review support that AMD is associated with increased risk of all-cause mortality. The evidence that AMD predicts incident CVD events or CVD mortality remains inclusive and warrants further study in the future.

## 1. Introduction

Age-related macular degeneration (AMD) is a major cause of irreversible vision loss among elderly people in the developed world [1, 2]. AMD has both early and late stages. It is affected by the dysfunction of a specialized cell layer in the back of the eye called the retinal pigmented epithelium (RPE). The macula has a high density of photoreceptors and provides detailed central vision [3]. The common features of early AMD include the presence of drusen and pigmentary abnormalities in the RPE; and late AMD is manifested through geographic atrophy or the development of neovascularization [3, 4].

Although the precise etiology of AMD remains unknown, the current evidence suggests that there are multiple similarities in the pathogenic mechanisms of both AMD and cardiovascular disease (CVD). AMD and CVD may connote shared underlying pathophysiological factors (e.g., advancing age, smoking, obesity, C-reactive protein, Apo lipoprotein E gene, and complement factor H) [3, 5]. But epidemiological studies have not found a consistent association of AMD and CVD [6–9], although a previous systematic review [10] including 13 population-based studies concluded that there is an association between AMD and CVD risk and suggested that AMD is predictive of a small increase in risk of future CVD. Thirteen cohort studies with a total of 1,593,390 participants with 155,500 CVD events (92,039: stroke and 62,737: CHD) were included in previous systematic review. However, the systematic review omitted several important papers. Furthermore, 5 of the 13 cohort studies included in that review used a retrospective design. In addition, this review did not investigate the link between AMD and risk of mortality. Therefore, we conducted a systematic review to better understand the role of AMD as a risk factor for CVD events and mortality.

## 2. Methods

### 2.1. Data Sources and Search Strategy

Preferred Reporting Items for Systematic Review and Meta-Analyses (PRISMA) statement was followed [11]. We first searched the literature in April 2014 of the Medline (Ovid) and Embase (Ovid) using the following search terms: “retina macula degeneration”, “retinal degeneration”, “Age-related macular degeneration”, “Cardiovascular Disease”, “coronary heart disease”, “heart failure”, and so on. To make sure our study was based on up-to-date results, we further updated the literature search of the Cochrane Library, Medline, and Embase on November 11, 2015. Besides that, additional studies were identified through the reference lists of relevant reports.

### 2.2. Study Selection and Data Extraction

Two investigators (Y. W and Y. J. X) independently screened the titles or abstracts, or both, of the search results and assessed the remaining full-text articles for eligibility. Any uncertainty regarding eligibility was resolved by discussion. Studies were eligible for our systematic review if (1) the exposure of interest was AMD; (2) the outcome of interest was cardiovascular events (coronary heart disease (CHD), myocardial infarction (MI), heart failure, stroke, or cardiovascular disease mortality) or all-cause mortality; and (3) the study was a population-based cohort study (i.e., not meeting abstracts or review articles). In the case of multiple publications from one study, we chose the articles with the largest sample or the longest follow-up interval.

Two investigators extracted the following data using standardized data extraction form: name of first author and year of publication; country cohort details and the number of participants; characteristic of the study population; mean follow-up years; assessment of exposure and outcome; type of CVD outcome; and covariate adjustment.

### 2.3. Data Analysis

We used the relative risk (RR) as the common measure of association across studies, and the hazard ratio (HR) or odds ratio (OR) was considered equivalent to the RR, while the OR was converted into RR by the formula RR = OR/[(1 − Po) + (Po × OR)], in which Po is the incidence of the outcome of interest in the nonexplosive group. Forest plots were produced to visually assess the RR and corresponding 95% confidence interval (CI) across studies [12]. The presence of heterogeneity across studies was evaluated by *Q* statistic (significance level of *P* < 0.10) and *I* ^2^ statistic (ranges from 0% to 100% with lower values representing less heterogeneity) [13]. The RRs were pooled using the DerSimonian and Laird inverse-variance-weighted random-effects models. What is more, we investigated the influence of a single study on the overall risk estimates by omitting any of the studies in each turn. We also conducted subgroup analyses stratified by number of participants (<10000 versus ≥10000), length of follow-up (<5 versus ≥5), type, and study design at baseline to assess the impacts of these variables on outcomes.

Analyses were performed with RevMan (version 5.2 for Windows; the Nordic Cochrane Centre, Copenhagen, Denmark). All statistical tests were 2-sided and *α* < 0.05 was considered statistically significant level.

## 3. Results

### 3.1. Literature Search

The search strategy resulted in 4,156 unique citations (Figure 3). Of these, we included 27 articles after review of the title or abstract. After detailed examination, 7 literatures were excluded (reasons shown in Figure 3). In total, 20 articles [6–9, 14–29] were included, and the full list of publications is shown in Table 1.

### 3.2. Study Characteristics

Table 1 presents study characteristic for included 20 cohort studies. These studies were published between 2002 and 2015. The sizes of cases diagnosed as AMD ranged from 181 to 167,838 (total 367,963). The sizes of participants ranged from 860 to 1,445,677 (total 2,985,316). There were 6 retrospective studies and 14 prospective studies. Three studies were conducted in late AMD only. The mean length of follow-up ranged from 2 to 13.6 years, with a median of 7.7 years. Some studies reported multiple outcomes.

### 3.3. AMD and Risk of Mortality

**Figure 1:**
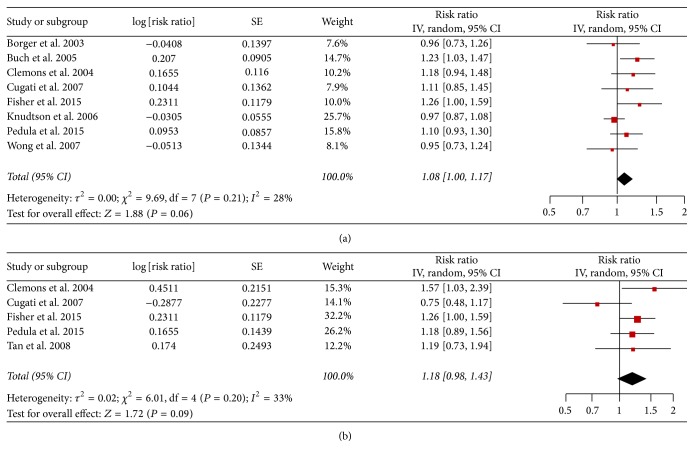
(a) Adjusted relative risks of all-cause mortality associated with AMD. (b) RR of cardiovascular disease mortality associated with AMD.

Each study and all studies reporting AMD and risk of mortality and cardiovascular disease mortality are shown in Figure 1.

For all-cause mortality, we identified 8 studies of AMD and risk of mortality incidence, involving 32,583 participants. Overall, there was a statistically significant increment of 8% among AMD patients (RR = 1.08, 95% CI: 1.00–1.17, Figure 1(a)) compared with those of non-AMD patients, and a low heterogeneity was detected (*I* ^2^ = 28%; *P* = 0.21).

For cardiovascular disease mortality we identified 5 studies of AMD and risk of cardiovascular disease mortality incidence, involving 17,250 participants (Table 1). The pooled RR (95% CI) was 1.18 (0.98–1.43; *I* ^2^ = 33.0%; *P* for heterogeneity = 0.20; Figure 1(b)).

### 3.4. AMD and Risk of Cardiovascular Events

**Figure 2:**
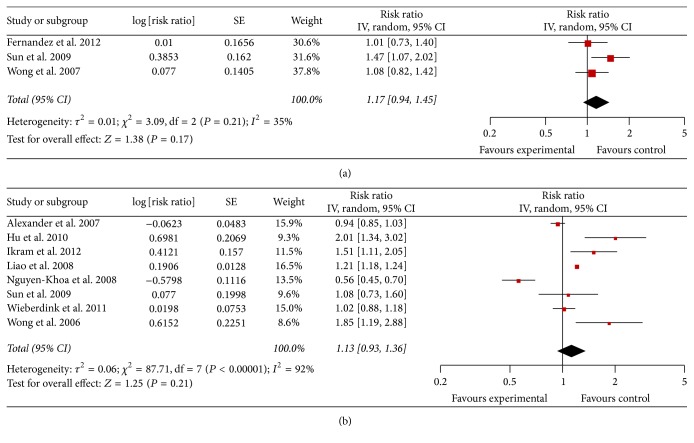
(a) RR of CHD associated with AMD. (b) RR of stroke associated with AMD.

**Figure 3:**
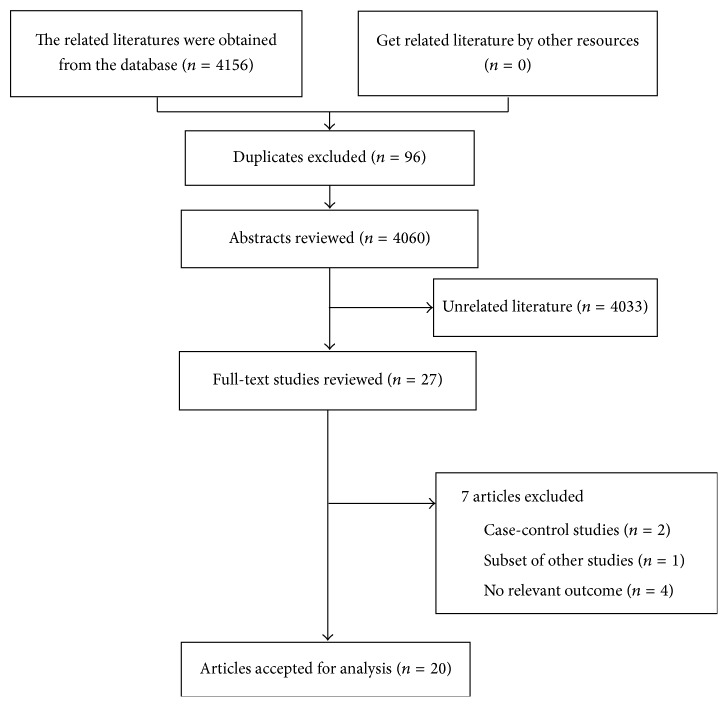
Flow chart of the meta-analysis of AMD and CVD.

Each study and all studies reporting AMD and cardiovascular events (CHD, MI, and stroke) are shown in Figure 2.

For CHD risk, we identified 3 studies of AMD and risk of CHD incidence, involving 18,353 participants (Table 1). Again, the pooled RR (95% CI) was 1.17 (0.94–1.45; *I* ^2^ = 35%; *P* for heterogeneity = 0.21; Figure 2(a)). And we identified 4 studies of AMD and risk of MI incidence, and high heterogeneity was detected (*I* ^2^ = 95%; *P* < 0.0001). Due to the statistically undetectable heterogeneity, meta-analysis for the MI was cancelled and a descriptive review was conducted instead. One study [18] reported a positive association (i.e., RR > 1.00), two studies [6, 21] reported RR < 1.00, which was not statistically significant and one study [26] reported a negative association (i.e., RR < 1.00).

For stroke risk, we identified 8 studies of AMD and risk of stroke incidence, involving 1,424,573 participants (Table 1). Four studies reported a positive association (i.e., RR > 1.00), three studies reported RR < 1.00 but this was not statistically significant and one study reported RR < 1.00 and it was statistically significant. High heterogeneity was detected (*I* ^2^ = 92%; *P* < 0.00001), and the RR (95% CI) from the random-effects model was 1.13 (0.93–1.36; Figure 2(b)).

### 3.5. Subgroup and Sensitivity Analyses

Sensitivity analyses were conducted to explore potential sources of heterogeneity in the association between AMD and stroke. Further analyses investigating the influence of a single study on the overall risk estimate by omitting any of the studies in each turn suggested the overall risk estimates were not substantially modified by any single study, with a range from 1.06 (95% CI: 0.87–1.29) to 1.15 (95% CI: 0.93–1.44). In addition, no single study substantially contributed to the heterogeneity across studies.

We conducted subgroup analyses by length of follow-up (<5 versus ≥5), number of participants (<10000 versus ≥10000), type, and study design (Table 2). Exploration of the effect by the type of AMD (early AMD/late AMD) suggested significant association between early AMD and risk of stroke (RR: 1.21, [95% CI: 1.03 to 1.42]; *P* = 0.007). The subgroup analysis by study design (i.e., prospective versus retrospective) and number of participants (<10000 versus ≥10000) did not suggest apparent difference.

## 4. Discussion

In the meta-analysis of 20 population-based cohort studies, our findings show that AMD is associated with around 8% increased risk of all-cause mortality. From the clinical view point, these values may be considerable. There was no evidence of between-study heterogeneity. We did not find a significant relationship between AMD and incident of cardiovascular events (CHD and stroke) and cardiovascular disease mortality, when data from both prospective and retrospective cohort studies were summarized. But heterogeneity between studies was high.

AMD is a chronic disorder and the major cause of permanent visual impairment among elderly people [1, 2]. Our meta-analysis of a significant trend of increased all-cause mortality with AMD suggests that AMD (early or late AMD) may reflect the status of systemic processes associated with biological aging, being a marker of underlying serious somatic factors or diseases, which could be associated with increased biological aging and decreased survival [4]. However, the precise mechanism of this relationship is unclear. Several plausible mechanisms for the relationship between AMD and mortality were shared common risk factors. It is possible that AMD accelerates the aging process through poorer vision and greater frailty, leading to more accidents, falls, and fractures, all of which have been linked to higher mortality [30, 31].

Several pathogenic mechanisms for the observed relationship of AMD with CVD have been proposed [1, 2]. Chronic inflammation is a plausible biologic mechanism for both AMD and CVD [4]. However, our meta-analysis is to show no association of AMD with MI incidence, stroke, and CHD events and cardiovascular disease mortality. The nonsignificant association between AMD and cardiovascular events and cardiovascular disease mortality may arise from several sources. Inconsistencies between studies might have arisen from the undocumented use of antiangiogenic therapy for the treatment of neovascularization in late AMD. Since the first antivascular endothelial growth factor (anti-VEGF) medication approved by the US Food and Drug Administration (FDA) for intravitreal use, pegaptanib, in 2006, the safety of anti-VEGF agents has been controversial given the potential for increased risk of atherothrombotic events when these agents are used systemically [32, 33]. Therefore, more studies confirming that a link exists between AMD and cardiovascular events would be needed before such recommendations can be made. In addition, in some of the studies found in our search, there was a lack of clinical validation to confirm the AMD diagnosis and no information about the confounding effect of smoking on the relationship.

For stroke, we observed substantial heterogeneity in those studies. This is not surprising given the variation in study designs and characteristics of populations between studies. Moreover, we found a significant association between early AMD and over 5 years of follow-up subgroup and risk of stroke incidence. High heterogeneity is observed within all subgroups. Our results and previous work on the topic highlighted the paucity of data regarding the predictive value of AMD in CHD/CVD events and likely reflect the heterogeneity in the study design, length of follow-up, and accuracy of the definitions of ocular and CVD outcomes used in the few studies addressing this clinical question. These differences may explain the discordance of results across studies. The assessment of differences in the prevalence of AMD among ethnicities likely requires a larger number of patients with sufficient power.

Several limitations should be acknowledged as well. First, significant heterogeneity was observed across studies for some outcomes, which may arise from differences in participants' characteristics, study designs, mean follow-up years, sample sizes, and analysis strategies. So we performed subgroup analyses on potential confounders. But moderate-to-high heterogeneities still remained in many subgroups. Second, our meta-analysis was limited to published reports. The literature screening and data extraction were conducted independently by two reviewers, and, thus, selection bias was unlikely. Third, the definition of AMD varied across studies. Among some studies, information on CVD was obtained by self-reporting from study participants. In addition, the associations were presented in different forms (OR, HR, RR, etc.), and RR was used as the common measure of association in this meta-analysis. The summary results might be influenced by the conversion of other measures into RR, but such influence, if any, is likely to be small because only a few studies reported ORs. Last, this study includes those inherent in retrospective database studies. The claims data lacks important health-related information such as height, weight, and smoking history. Despite strong associations, residual confounding is still possible given that many studies did not adjust lifestyle factors (e.g., smoking, unhealthy diet, and exercise) in their models.

## 5. Conclusions

Findings from this systematic review support that AMD is associated with increased risk of all-cause mortality. The evidence that AMD predicts incident CVD events or CVD mortality remains inclusive and warrants further study in the future.

## Figures and Tables

**Table 1:** Characteristics of 20 prospective cohort studies of AMD and risk of cardiovascular disease outcomes included in this meta-analysis.

Study: first author, year	Country cohort details	Participants, age (years)	Early AMD	late AMD	Eligibility criteria	Mean follow-up, years	Outcome	Adjustment for covariates	Study design
Borger et al. 2003 [14]	The Rotterdam Study (Netherlands)	6339, age >55	477	104	Retinal photograph	7	Mortality (medical records)	Age, gender, smoking, BMI, cholesterol level, atherosclerosis, hypertension, history of cardiovascular disease	prospective
Buch et al. 2005 [15]	The Copenhagen City Eye Study (Denmark)	866, 60–80	228	31	Retinal photograph	14	Mortality (ICD8, ICD10)	Age, gender, smoking status, alcohol consumption, BMI, total cholesterol level, hypertension, cardiovascular disease, diabetes mellitus, any cataract, visual loss (20/40)	prospective
Clemons et al. 2004 [16]	AREDS	4753, 55–81	2650	957	Fundus photograph	6.5	Mortality (medical records)	Age, sex, race, education, smoking status, BMI, diabetes mellitus, angina, cancer, hypertension	prospective
Cugati et al. 2007 [17]	BMES (Australian)	3532, age > 49	177	72	Retinal photograph	10.7	Mortality (ICD9, ICD10)	Age, sex, BMI, hypertension diabetes mellitus, current smoking, and baseline history of angina, acute myocardial infarction, triglyceride level, fibrinogen level, educational level, walking disability, stroke	prospective
Duan et al. 2007 [18]	USA	1445677, mean 76 (7.8)	126693	30885	ICD-9	2	MI (ICD-9)	Age, gender, race, hypertension, and diabetes	retrospective
Fernandez et al. 2012 [19]	MESA (USA)	6233, 45–84	893	27	Retinal photograph	5.4	CHD and CVD (medical records)	Age, sex, race, hypertension, cigarette smoking, site of enrollment, CRP, level of education and diabetes, BMI, serum total cholesterol, low-density lipoprotein cholesterol	prospective
Fisher et al. 2015 [20]	AGES (Israel)	4910, 67–96	1064	277	Retinal photograph	8.6	Mortality (ICD-9)	Age, gender	prospective
Golan et al. 2011 [21]	MHS (Israel)	68217, age ≥ 65	6546		ICD-9	4.75	MI (ICD-9)	Age, gender	retrospective
Hu et al. 2010 [22]	LHID (Taiwan)	1254, mean 62.7	0	209	ICD-9	5	Stroke (ICD-9)	Age, gender, monthly income, level of urbanization, the geographic region of the community	retrospective
Ikram et al. 2012 [23]	ARICS (USA)	12216 45–64	576	15	Retinal photograph	13	Stroke (medical records)	Mean arterial blood pressure, antihypertensive medications, cell count fasting glucose, white blood, total cholesterol, HDL-cholesterol, triglyceride levels, BMI, atrial fibrillation, cigarette smoking, alcohol consumption status.	prospective
Knudtson et al. 2006 [24]	BDES (Australian)	4747, 43–84	909	79	Retinal photograph	13.2	Mortality (ICD-9, ICD-10)	Age, sex, proteinuria, history of cancer, BMI, BMI2, ratio of total to high-density lipoprotein cholesterol level, smoking, pulse rate, education, diabetes status, cardiovascular SBP disease history, sedentary lifestyle	prospective
Liao et al. 2008 [25]	CMS (USA)	1303186, ≥65.0	140645	27193	ICD-9	2	Stroke (ICD-9)	Age, gender, race, hypertension, diabetes	retrospective
Nguyen-Khoa et al. 2008 [26]	USA	27411, 50–95	0	7203	ICD-9	3.5	MI and CVD (ICD-9)	Presence of angina, cardiac arrhythmia, Charlson score, congestive heart failure, diabetes, heart disease, history of acute MI, history of CVA, hyperlipidemia, hypertension, other cerebrovascular diseases	retrospective
Pedula et al. 2015 [27]	SOF (USA)	1202, ≥65	441	46	Retinal photograph	9.5	Mortality and CVD (ICD-9)	Age, race, self-reported frailty, body mass index, Minimental State Examination score, walking speed, history of congestive heart failure, history of myocardial infarction, history of chronic obstructive pulmonary disease, history of thiazide diuretic use	Prospective
Sun et al. 2009 [28]	CHS (USA)	1715, 69–97	268	24	Retinal photograph	6	CHD and stroke (medical records)	Age, gender, race, systolic and diastolic, blood pressure, hypertension status, fasting glucose, triglyceride, low-density lipoprotein cholesterol, cigarette smoking, pack-years of smoking, CRP	prospective
Tan et al. 2008 [29]	BMES (Australian)	2853, 49–97	130	51	Retinal photograph	11	Mortality (ICD-9, ICD-10)	Age, gender, hypertension, diabetes, smoking, BMI, serum lipids, fibrinogen, white cell count	prospective
Wieberdink et al. 2011 [9]	The Rotterdam Study (Netherlands)	6207, 61.7–74.8	2166	93	Retinal photograph	13.6	Stroke (medical records)	Age, sex, diabetes, systolic blood pressure, antihypertensive medication, current smoking, total cholesterol, high-density lipoprotein cholesterol, carotid artery plaques, body mass index, alcohol intake and CRP, APOE, CFH	prospective
Wong et al. 2006 [8]	ARICS (USA)	10405, 49–73	498	10	Retinal photograph	8	Stroke (ICD-9, ICD-10)	Age, sex, ethnicity, site, systolic blood pressure, diabetes, antihypertensive medication use, cigarette smoking	prospective
Wong et al. 2007 [7]	ARICS (USA)	11414, 49–73	540	15	Retinal photograph	8	Mortality and CHD (medical records)	Age, gender, race, and center, and then further for education, body mass index, systolic and diastolic BPs, diabetes status, total plasma cholesterol and HDL-cholesterol, triglyceride, glucose, pack-years of cigarette smoking, current alcohol consumption	prospective
Alexander et al. 2007 [6]	USA	62179, mean 80.5	0	15771	ICD-9	2	MI and CVD (ICD-9)	Age, race, gender, length of data within the database	retrospective

AREDS: Age-Related Eye Disease Study; BMES: Blue Mountains Eye Study; MESA: Multiethnic Study of Atherosclerosis; AGES: Age, Gene/Environment Susceptibility Reykjavik Study; LHID: Longitudinal Health Insurance Database; ARICS: Atherosclerosis Risk in Communities Study; BDES: Beaver Dam Eye Study; CMS: Medicare and Medicaid Services; SOF: Study of Osteoporotic Fractures; CHS: Cardiovascular Health Study; BMI: body mass index.

**Table 2:** Stratified analyses of stroke associated with AMD.

Group	Number of studies	RR (95% CI)	*P* (heterogeneity)	*I* ^2^ (%)
Total	8	1.13 [0.93, 1.36]	<0.00001	92
Length of follow-up (years)				
<5	3	0.88 [0.64, 1.20]	<0.00001	97
≥5	5	1.40 [1.05, 1.87]	0.002	76
Number of participants				
<10000	3	1.27 [0.86, 1.87]	0.009	79
≥10000	5	1.07 [0.83, 1.38]	<0.00001	95
Type				
Early AMD	5	1.21 [1.03, 1.42]	0.007	71
Late AMD	6	1.14 [0.85, 1.52]	<0.00001	95
Study design				
Prospective	4	1.07 [0.90, 1.26]	0.03	66
Retrospective	4	1.22 [0.75, 1.99]	<0.00001	95
